# Patients' Experiences and Attitudes of Using a Secure Mobile Phone App for Medical Photography: Qualitative Survey Study

**DOI:** 10.2196/14412

**Published:** 2020-05-12

**Authors:** Kirk D Wyatt, Anissa Finley, Richard Uribe, Peter Pallagi, Brian Willaert, Steve Ommen, James Yiannias, Thomas Hellmich

**Affiliations:** 1 Division of Pediatric Hematology/Oncology Department of Pediatric and Adolescent Medicine Mayo Clinic Rochester, MN United States; 2 Department of Emergency Medicine Mayo Clinic Rochester, MN United States; 3 Information Technology Mayo Clinic Scottsdale, AZ United States; 4 Media Support Services Mayo Clinic Scottsdale, AZ United States; 5 Information Technology Mayo Clinic Rochester, MN United States; 6 Center for Connected Care Mayo Clinic Rochester, MN United States; 7 Department of Dermatology Mayo Clinic Scottsdale, AZ United States

**Keywords:** photography, mobile apps, telemedicine, electronic health records, mobile phone, digital imaging, dermatology, vascular medicine, family medicine

## Abstract

**Background:**

Point-of-care clinical photography using mobile devices is coming of age as a new standard of care for clinical documentation. High-quality cameras in modern smartphones facilitate faithful reproduction of clinical findings in photographs; however, clinical photographs captured on mobile devices are often taken using the native camera app on the device and transmitted using relatively insecure methods (eg, SMS text message and email) that do not preserve images as part of the electronic medical records. Native camera apps lack robust security features and direct integration with electronic health records (EHRs), which may limit patient acceptability and usefulness to clinicians. In March 2015, Mayo Clinic overcame these barriers by launching an internally developed mobile app that allows health care providers to securely capture clinical photographs and upload them to the EHR in a manner that is compliant with patient privacy and confidentiality regulations.

**Objective:**

The study aimed to understand the perceptions, attitudes, and experiences of patients who were photographed using a mobile point-of-care clinical image capture app.

**Methods:**

The study included a mail-out survey sent to 292 patients in Rochester, Minnesota, who were photographed using a mobile point-of-care clinical image capture app within a preceding 2-week period.

**Results:**

The surveys were completed by 71 patients who recalled being photographed. Patients were seen in 18 different departments, with the most common departments being dermatology (19/71, 27%), vascular medicine (17/71, 24%), and family medicine (10/71, 14%). Most patients (49/62, 79%) reported that photographs were taken to simply document the appearance of a clinical finding for future reference. Only 16% (10/62) of patients said the photographs were used to obtain advice from a specialist. Furthermore, 74% (51/69) of the patients said they would recommend medical photography to others and 67% (46/69) of them thought the photos favorably affected their care. Patients were largely indifferent about the device used for photography (mobile device vs professional camera; 40/69, 58%) or the identity of the photographer (provider vs professional photographer; 52/69, 75%). In addition, 90% (64/71) of patients found reuse of photographs for one-on-one learner education to be acceptable. Acceptability for other uses declined as the size of the audience increased, with only 42% (30/71) of patients deeming reuse on social media for medical education as appropriate. Only 3% (2/71) of patients expressed privacy or confidentiality concerns. Furthermore, 52% (33/63) of patients preferred to provide consent verbally, and 21% (13/63) of them did not think a specific consent process was necessary.

**Conclusions:**

Patient attitudes regarding medical photography using a secure EHR-integrated app were favorable. Patients perceived that photography improved their care despite the most common reason for photography being to simply document the appearance of a clinical finding for future reference. Whenever possible, health care providers should utilize secure EHR-integrated apps for point-of-care medical photography using mobile devices.

## Introduction

### Clinical Photography

Clinical photography has been standard practice in the fields of dermatology [1,2], plastic surgery [1,3] and dentistry [4] for years, and it has emerged as a useful tool for use by general practitioners [5] and emergency medicine providers [6] as well. Clinical photographs can be captured by professional medical photographers using clinic-owned equipment or by health care providers themselves using a clinic-owned camera or their personal mobile device. With personal smartphone ownership approaching nearly 3 billion, the usage of mobile devices is expected only to increase [7].

Clinical photography has many uses, and most patients seem to find it useful in the course of their care [8,9]. It can provide more vivid descriptions than a provider could detail in their written documentation, allow providers to follow progression of a disease over time, facilitate telemedicine, and allow patients or their caregivers to see clinical findings they might not otherwise be able to see themselves (eg, finding on back or intraoperative findings) [10,11].

### Privacy and Confidentiality Concerns

A number of legal and ethical issues concerning patient privacy and data security arise in the course of clinical photography; for example, patients may feel uncomfortable with clinical photography that involves sensitive areas (eg, genitalia) [6]. Patient attitudes may vary when a professional-grade, clinic-owned camera is used compared with a health care provider’s personal mobile phone [8,9,12,13]. Consent practices may vary [8], and the optimal method of consent (ie, implied, verbal, or written) for medical photography is unclear [2,9]. Furthermore, when patients consent to medical photography, they may assume that the photographs will only be used to provide clinical care, yet it is clear that clinical photographs may be beneficial tools for medical education [10]. The extent to which patients are comfortable with photographs being used for various educational purposes may be variable [6,8,9,12,13]. Although patients are, in general, accepting of reuse for medical care and education in small settings, they are less accepting of reuse in media that are distributed to larger audiences and the lay public (eg, social media) [6,8,9,12,13].

Legal provisions in the United States and elsewhere dictate the manner in which health care providers must keep protected health information private and secure. Although some health care providers transmit clinical photographs to colleagues using methods that lack certain privacy and security features, such as text messaging or email [2], various electronic health record (EHR) vendors have incorporated secure clinical photography modules that include patient identity management and security features within the mobile apps. Despite this, these secure mobile apps may be relatively underused. In a Canadian survey of neurosurgical residents, 45% of whom used their smartphone for clinical photography, 89% stored photos or videos of patients in the native smartphone app and only 8% used a password-protected app [9]. More concerning is that only 32% of users deleted photographs immediately after use, and 23% of them said that they do not routinely delete patient photographs on their phones [9]. At the same time, a survey of dermatologists—approximately half of whom used a smartphone for clinical photography—revealed that only 43% used a secure smartphone app that is integrated with the EHR [14]. This practice is problematic for several reasons. First, mobile devices may not be uniformly password protected to prevent unauthorized access. Second, mobile devices often automatically backup photos to a personal cloud storage service (eg, iCloud, Apple Inc; DropBox, Dropbox, Inc). These tools may not be compliant with health care data protection laws as they may lack sufficient security to prevent unauthorized viewing and there may be no reliable mechanism to ensure that patients are notified in the event of a data breech on the cloud storage service. Finally, capturing a photograph using a native app implies that the photograph will not become part of the medical record. Not only does this limit visibility by other clinicians participating in the patient’s care, but it also implies that the photograph may be transmitted using insecure methods (eg, personal email, text message).

### Goal

In March 2015, after legal and policy review, Mayo Clinic released an internally developed iOS-based clinical photography app named PhotoExam. The app permits any member of the health care team with clinical documentation privileges to capture photographs using their personal mobile device or a clinic-owned device and directly upload the images to the EHR. The app’s features include patient identity management, Health Insurance Portability and Accountability Act (HIPAA) compliance, and confirmation that patient consent has been obtained before photography takes place. We previously reported app uptake at the 3 Mayo Clinic campuses in Minnesota, Florida, and Arizona as well as the regional Mayo Clinic Health System [11]. Considering that the existing literature largely focused on patient attitudes about medical photography performed either using a clinic-owned professional camera or a smartphone app that lacks security features and is not integrated with the EHR, we aimed to understand how patient experience may differ with PhotoExam.

Therefore, to assess perceived patient benefit, acceptability, and privacy/security concerns, we surveyed patients who were photographed by their health care providers using the PhotoExam app.

## Methods

### PhotoExam App

The PhotoExam app is an internally developed iOS-based mobile app that allows health care providers to take clinical photographs of patients and incorporate them into the EHR in a manner that is secure and HIPAA compliant. Providers launch the app either from the patient’s chart opened within a third-party EHR mobile app or they open the app from their home screen and then manually enter the patient’s medical record. A hard-stop requires providers to verify that they have obtained patient consent, according to departmental policies, before capturing photographs. Providers then select the anatomical site(s) they will photograph and use the smartphone camera to capture up to 6 photographs per anatomical site. Photos can be immediately deleted or retaken if quality is suboptimal. After all images have been captured, the app uploads the images to the patient’s medical record and provides confirmation that the images have been successfully uploaded. The photographs are then automatically deleted from the user’s device after upload is complete or whenever the user closes the app—whichever comes first. Images are never made accessible outside of the PhotoExam app (eg, within the native photo gallery). Newer versions of PhotoExam allow capture of short video clips; however, video recordings were not considered for this study.

### Patient Selection

We included a random sample of 300 adult patients or parents of pediatric patients (ie, aged <18 years) who were photographed using the PhotoExam app at Mayo Clinic, Rochester, Minnesota, within a preceding 2-week period and for whom a mailing address was available. Patients who refused research participation were excluded. We also excluded patients whose primary language was not English because the survey was written in English. To protect pediatric patients, we reviewed the photographs and clinical records of pediatric patients identified for potential inclusion and excluded patients who were seen for confidential visits or visits of a potentially emotionally sensitive nature. The excluded pediatric patients were substituted with another randomly selected pediatric patient (ie, aged <18 years) who had been photographed during the same 2-week period. 

### Survey Description

The survey included the patient’s name, medical record number, and the date of the most recent clinical visit where a health care provider photographed them using the PhotoExam app. Surveys were mailed to patients at their home address. Surveys corresponding to patients younger than 18 years were addressed to the patient’s parent or guardian. Patients who did not recall being photographed were asked to not answer further questions and return the survey. Questions generally related to the reason photos were taken, effect on timeliness of care, comfort level with medical photography, and the manner in which consent to photography was obtained. Patients were also allowed to share general comments or concerns about the practice of medical photography using mobile devices in free-text form (Multimedia Appendix 1). Patients who did not respond to the initial survey were sent a follow-up survey.

### Statistical Analysis

Responses are reported in counts and percentages with continuous variables summarized as means or medians and standard deviations or ranges, as appropriate. In cases where patients did not respond to individual questions, percentages represent the number of patients who responded to an individual question.

### Human Subjects Protection

The study procedures were approved by the Mayo Clinic Institutional Review Board.

## Results

### Patient Selection

**Figure 1:**
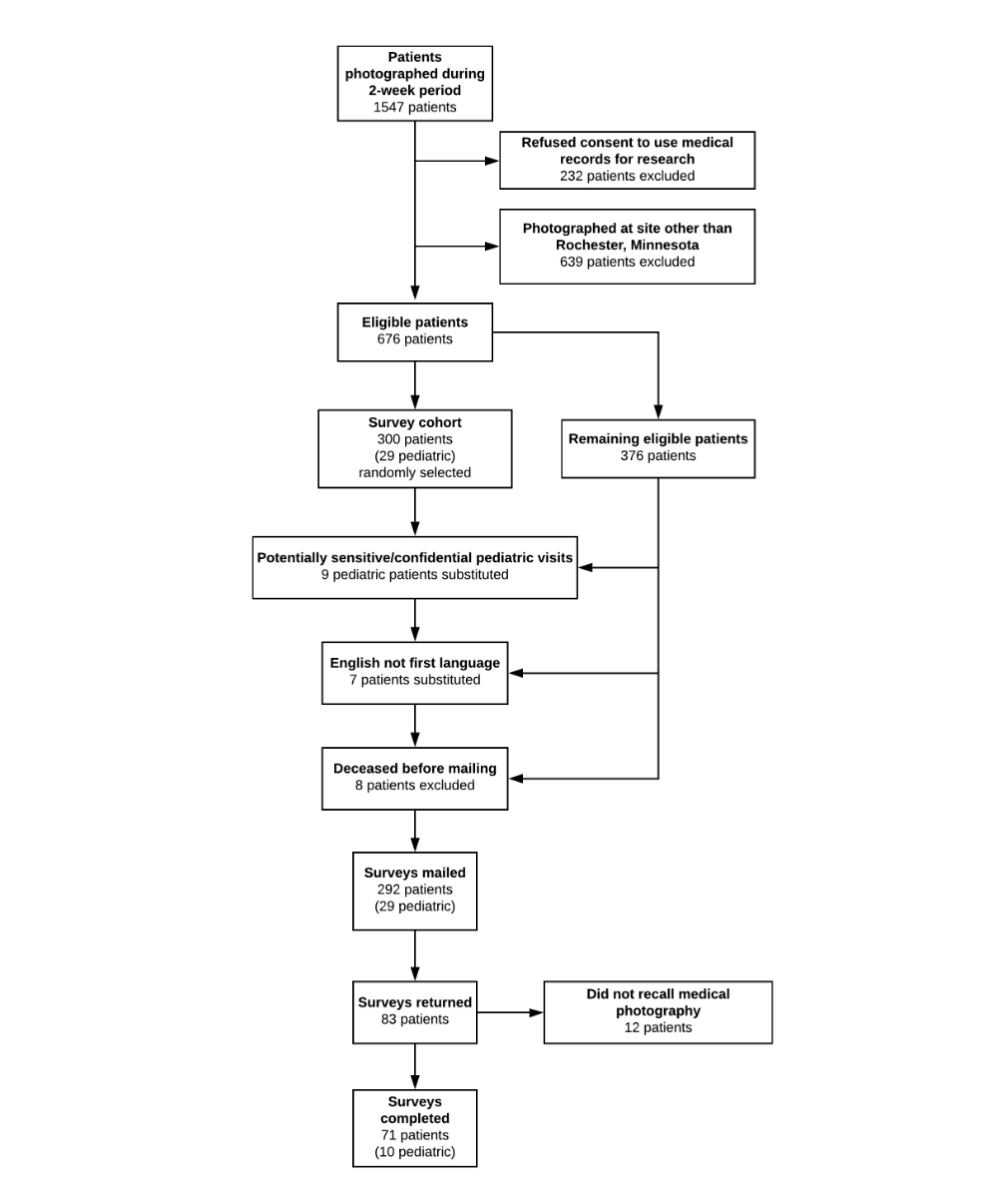
Patient selection.

Patients (n=1547) who were photographed with the app within a 2-week period were identified. After ineligible patients were excluded, surveys were printed and mailed to 292 patients, 29 of whom were pediatric patients (Figure 1). Surveys were mailed 51 days after the end of the 2-week period when photos were taken. A total of 64 completed surveys were returned after the initial mailing. The second mailing was sent to nonrespondents 1 month after the initial mailing (81 days after the end of the 2-week period where photos were taken). A total of 83 total surveys were returned. In addition, 14% (12/83 of respondents willing to complete the survey) adult patients did not recall a health care provider using a mobile device to take a photograph during a visit and therefore did not complete the remainder of the survey. The remainder of respondents (n=71; 10 pediatric) recalled medical photography taking place and completed the remainder of the survey.

### Population and Demographics

The patient demographics are shown in Table 1. Patients were seen in 18 different departments, with the most common departments being dermatology (19/71, 27%), vascular medicine (17/71, 24%), and family medicine (10/71, 14%).

**Table 1:** Patient demographics (N=83 surveys returned).

Characteristics		Values
Age (years), median (SD)		63 (23)
Younger than 18 years of age, n (%)		10 (12)
**Marital status (N=83 surveys returned), n (%)**		
	Single	19 (23)
	Married	59 (71)
	Divorced	4 (5)
	Widowed	1 (1)
**Race (N=83 surveys returned), n (%)**		
	White	82 (99)
	Other	1 (1)
Number of photos taken of patient using PhotoExam during an encounter, median (range)		2 (1-9)
Number of photos ever taken of patient using PhotoExam, all encounters included, median (range)		5 (1-164)
Days between clinical visit and survey completion (days), median (range)		68 (58-224)
**Eligible surveys (N=292 surveys mailed), n (%)**		
	Survey returned	83 (28.4)
	No response after 3 mailings	177 (60.6)
	Refused	24 (8.2)
	Deceased (survey not sent)	8 (2.7)
	Survey returned without Health Insurance Portability and Accountability Act authorization	3 (1.0)
	Deceased (notified after survey sent)	2 (0.6)
	Invalid mailing address	1 (0.3)
	Physically/mentally unable to complete	1 (0.3)
	Returned blank survey	1 (0.3)

### Rationale for Photography

Patients reported that the majority of photos were taken to document the appearance of the area for future reference (49/71, 69%). Furthermore, 14% (10/71) photos were taken to send to a specialist for review. Three percent (2/71) reported that photographs were taken for educational purposes, and the remainder were unsure why photographs were taken.

### Effect of Photography on Time to Diagnosis and Treatment When Specialist was Consulted

Two of the patients whose providers photographed them to obtain a specialist’s assistance with making the diagnosis said the photography had no effect on the time to diagnosis and treatment, but the remaining 3 indicated that it either slightly or significantly expedited time to diagnosis and treatment.

### Consent for Photography

Overall, 91% (60/66) of patients who were surveyed recalled being asked permission to take photographs, with three-fourth (45/60) of these patients providing verbal permission and the rest (15/60) providing written permission.

Of the patients who provided written consent, 53% (8/15) indicated that they only read a part of the consent form and 1 patient (1/15, 7%) did not read any part of the form, with the remaining 33% (5/15) patients indicating that they read the entire consent form word for word.

When patients were asked their preference for providing consent, 21% (13/63) did not think specific permission was necessary, 52% (n= 33/63) preferred verbal consent, and 27% (17/63) preferred to provide written consent, with 59% (10/17) of these patients requesting to provide written consent by signing the screen on the device used to take photos (a feature that was not available).

### Attitudes Regarding Photography Practice and Process

**Figure 2:**
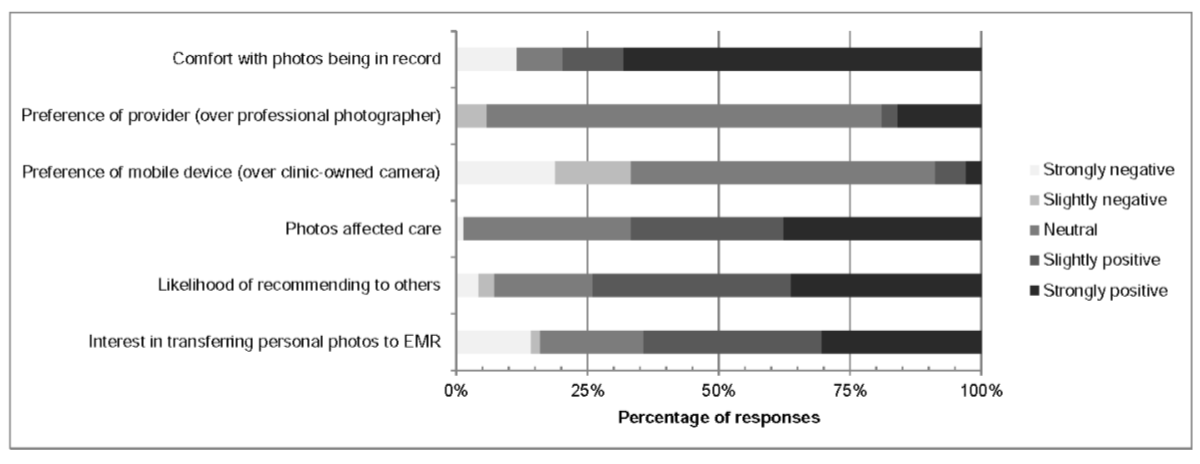
Attitudes regarding photography practice and process. EMR: electronic medical record.

Patients were asked a variety of questions regarding their attitudes about photography, including their comfort with photos being present in the EHR, preference of photographer, and preference of photography equipment. Patients were also asked whether photography affected their care and whether they would recommend photography to family or friends in a similar clinical scenario. Responses are shown in Figure 2, where Likert scale responses are color coded. Overall, 74% (51/69) of patients said they were likely to recommend mobile point-of-care clinical photography to others, and 67% (46/69) of patients thought the photos favorably affected their care.

Furthermore, 80% (56/70) of patients indicated that they owned a phone with a camera, and the majority of them expressed an interest in transferring personally taken photographs into the EHR (Figure 2). Three of the patients (3/55, 5%) who owned a smartphone experienced a provider taking a photograph of a phone screen to transfer a photograph from a personal device to the EHR.

### Privacy and Data Security Concerns

In total, 2 patients (2/71, 3%) indicated that they had concerns regarding privacy or confidentiality related to the photos. When asked for further explanation, one of these patients inquired whether the photos remain on the provider’s phone and another explained that they believed the photos were used professionally but that they did not know the provider who took the photos. Therefore, it appeared that these concerns did not represent a belief that a privacy breech had actually occurred.

In addition, 26% (18/70) of patients recalled that security features of the app were explained by the health care provider. Furthermore, 16% (11/70) indicated that security features were not explained, and the majority (41/70, 59%) of them did not remember or were unsure if security features were explained to them.

### Reuse of Photographs for Educational Purposes

Patients were asked about their comfort level with the photos that were taken being shared in various settings, provided that any personally identifying information was removed. In general, patients were supportive of use for medical education; however, comfort declined with progressively increasing audience sizes (Table 2).

**Table 2:** Patient comfort with reuse of photographs for educational purposes (N=71)

Reuse of photographs	Value, n (%)
One-on-one learner education	64 (90)
Large group (eg, classroom) medical learner education	57 (80)
Presentation at a national medical professional meeting	52 (73)
Publication in a medical journal or textbook	48 (68)
Publication on social media for medical education	30 (42)

### Free-Text Responses

Overall, 41% (29/71) of patients provided additional written comments regarding their experiences with being photographed using a mobile device. The comments included the following:

- Requests to have a personal copy of the photographs sent.
- Indication that answers regarding comfort with the photos would have been different had the anatomical site photographed been a more sensitive area.
- Indication that the ability to have medical photographs taken prevented the need for an additional office visit.
- General questions regarding the security of the app and desire for a patient education handout regarding the privacy and security of the app.
- Indication that the ability to review images allowed patients to track the progress of their medical condition.

## Discussion

### Principal Findings

To the best of our knowledge, this is the first report to focus on the attitudes and experiences of patients who were photographed using a secure EHR-integrated smartphone app designed specifically for medical photography. Patients were largely satisfied with clinical photography using the app, with 74% (51/69) of patients indicating they were likely to recommend a friend or family member to give permission for medical photography using a mobile device in a similar clinical situation. In addition, 67% (46/69) of patients perceived that the app’s use improved the care they received even though most patients indicated that the photograph was taken only to document the appearance of a clinical finding that could be referenced at a future visit. This suggests that patients see benefit in the documentation of findings in a manner that may facilitate future clinical care. Indeed, in free-text responses, 2 patients shared that it helped them to track the progress of their medical condition. Although a minority of photographs were sent to a specialist, some patients shared that sending photographs to a specialist prevented them from needing an additional office visit—presumably because of the use of telemedicine.

Responses demonstrated that some patients had a desire to incorporate personally taken photographs into their medical record and revealed evidence that some health care providers had resorted to using PhotoExam to take a photograph of a photograph displayed on a patient’s smartphone screen as a workaround to incorporate these photographs. EHR vendors should consider how to better integrate photograph upload functionality into their patient-facing products so that patient-taken photographs may complement those taken by providers. After the survey was administered, Mayo Clinic implemented a new commercially vended EHR that included the ability of patients to upload clinical photographs to providers with whom they have an established relationship. In the future, we will explore whether patients could be permitted to upload photographs to providers who are not already members of their care team (eg, emergency medicine provider).

The consent processes were variable. Although many patients feel that verbal consent is adequate, verbal consent processes may be problematic in that they may not elicit specific permission for all potential reuses (ie, medical education). Furthermore, even though written consent forms may ask for permission to use photographs for medical education, they may not explicitly detail various medical education uses for which patients may consent (eg, internal use one-on-one with learners vs widely distributed patient education pamphlet) and may simply include a boilerplate legal *blanket statement* seeking permission for all possible uses. We are currently evaluating the use of a 3-tiered consent form with clear language, allowing patients to consent for use of photographs for (1) clinical care only; (2) clinical care and internal education; or (3) clinical care, internal education, and external education.

### Comparison With Prior Work

In our survey, most patients who recalled the consent process indicated that they provided verbal consent, and the majority of patients felt that verbal consent was adequate. In contrast, a French survey involving 158 adult patients and parents of 114 pediatric patients photographed in a dermatology clinic observed that 80% of adult patients and 89% of parents thought written consent was necessary for medical photography [8]. Another study surveyed a convenience sample of 398 patients seen in dermatology clinics in New York City and observed that 78.4% (312/398) of respondents preferred to provide written consent compared with 14.1% (56/398) who preferred verbal consent. A similar study conducted in Chicago reported 58.7% (172/293) preferring written consent over verbal consent (113/293, 38.6%) [13]. Potential explanations for the differences observed between our survey and those reported in the literature include temporal changes in patient attitudes between the survey time points (with patients gaining acceptability over time); patients’ understanding of security features in the PhotoExam app; and sociodemographic differences between study populations, including the enrichment of our community with health care workers.

Patients were overwhelmingly supportive of reuse of their clinical photographs for educational purposes. Acceptability of different uses decreased as the size of the audience increased. These observations were consistent with other studies that generally reported high rates (ie, >80%) of acceptability of use of photographs for medical teaching and less comfort with widespread distribution to the lay public [6,8,9,12,13]. Despite these high levels of acceptability of image reuse, consent for reuse should always be sought (either at the time of photography or at the time reuse is desired).

We also observed, consistent with other studies, that comfort with photographs depends on the anatomical area photographed, with patients expressing discomfort with photography of sensitive anatomic areas [8,9]. A total of 3% (2/71) of actual photos in our sample were of the breast, buttock, or genitalia, and free-text comments suggested that other patients would have expressed more discomfort if they had been photographed in a more anatomically sensitive area.

More than half of patients had no preference regarding whether a provider’s personal mobile device was used for clinical photography compared with a clinic-owned camera. This is in contradiction to multiple previous studies that reported poor acceptability of providers’ personal mobile devices for clinical photography [8,9,12,13]. As with the differences observed regarding attitudes about the consent process in other studies, it is unclear if changes in patients’ perceptions over time, the security features of the PhotoExam app, or sociodemographic differences account for the inconsistency between studies.

### Strengths and Limitations

This study has several strengths. First, we included a random sample of pediatric and adult patients seen in a variety of departments who were photographed using a mobile app designed specifically for the purpose of clinical medical photography. Surveys were mailed shortly after the clinical encounter where photography occurred to maximize recall. In addition, we surveyed patients across multiple domains, including the perceived benefit, attitudes regarding use of photographs for education, preferred method of providing consent, and preferred camera type. We were also able to incorporate additional information from the medical record, including patient demographics, anatomical site(s) photographed, and the department of the specialist who photographed the patient.

Conversely, there are several limitations to this study. Although initial surveys were sent within 10 weeks of the occurrence of medical photography and most completed surveys were filled out promptly, we were surprised that 14% (12/82) of those who returned the survey did not recall that they were photographed by their health care provider. This significantly limited our ability to gather information about these patients’ experience with medical photography. It is unclear whether patients’ failure to recall the photography is evidence that medical photography was viewed as an insignificant and unimportant part of their visit or whether it was perceived as standard of care and therefore not memorable. Although our response rate was low, at 28% (83/292), this was similar to response rates of similar internal patient surveys conducted at Mayo Clinic. The low response rate led to a small sample size, which limits the generalizability of the results and limits our ability to make meaningful conclusions regarding questions that only a subset of patients were eligible to respond to based on other responses.

In addition, responses were only gathered from patients, and clinical outcome measurement was limited to a patient’s perception of how medical photography affected their care. Use of a formally validated survey would improve the internal and external validity of the study. Health care providers who use clinical photographs may have other insights into how clinical photography affects clinical management. To address provider experience, we plan to separately report a survey of health care providers who have used PhotoExam.

It is also important to note that our sample is biased in that we only surveyed patients who were photographed using the PhotoExam app. By nature of permitting a health care provider to photograph them with the app, patients may have preconceived notions of how the app may be beneficial or may have different attitudes regarding medical photography when compared with patients who refused medical photography with the PhotoExam app. In the authors’ personal experience, refusal of consent to photograph is rare and is generally in the context of a request to photograph an anatomically sensitive area. As noted above, the patient population seen in Rochester, Minnesota, may not be representative of the broader United States. Although our population includes both local patients and those who travel from elsewhere to receive care, our local population is enriched with health care workers, whose attitudes may differ from those who do not work in health care; for example, 82% (68/83) of patients in our study were from Minnesota, with 47% (39/83) of included patients residing in Rochester, Minnesota, and an additional 7% (6/83) living outside of Rochester, Minnesota, but within Olmsted County. In addition, our patient population was 99% (82/83) white. A more racially diverse cohort may have resulted if we had provided translated surveys for non–English-speaking patients. Owing to this limitation, our current results may have failed to reflect important cultural differences in attitudes that may vary among patients.

We were encouraged that, in general, patients felt that medical photography using the app favorably affected their care, and only on a rare occasion did patients perceive it to be detrimental to their care. Concerns about privacy were rare, and patients were generally comfortable with clinical photography conducted using their provider’s mobile device. These findings suggest that PhotoExam was implemented in a way that facilitates patient care and is sensitive to patients’ privacy and confidentiality.

### Conclusions

In summary, the PhotoExam app was well received by patients, addressed privacy and confidentiality issues, and was perceived to favorably affect patient care. We discovered no major patient-perceived barriers to implementing point-of-care clinical photography. In this regard, point-of-care medical photography is an up-and-coming best practice that we predict will become a new standard of care because of the obvious clinical benefit and ease with which photographs can be captured and entered into the medical record [15]. Future priorities include explicitly clarifying permissible educational reuses of photographs using uniform consent processes.

## Appendix

Multimedia Appendix 1 Patient survey.

## Abbreviations

EHR: electronic health record
HIPAA: Health Insurance Portability and Accountability Act
