# Supplementary material for: Patients' Experiences and Attitudes of Using a Secure Mobile Phone App for Medical Photography: Qualitative Survey Study
Source: J Med Internet Res. 2020 May 12;22(5):e14412. doi: 10.2196/14412 (PMC7251478; doi:10.2196/14412)
Supplement: Multimedia Appendix 1 [file jmir_v22i5e14412_app1.pdf]

MAYO CLINIC

Patient Survey

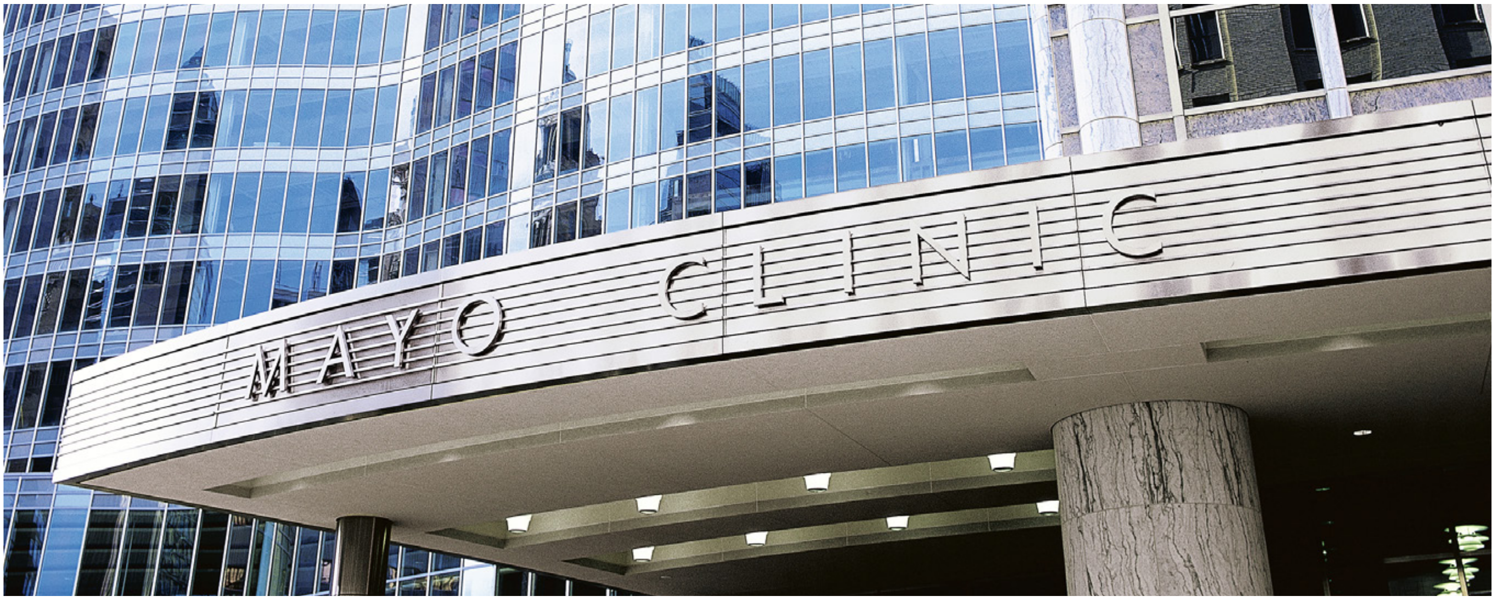

Survey Research Center

1-8

9-16

Please enter above any missing information or change any that is incorrect.

INSTRUCTIONS: PLEASE CHECK THE APPROPRIATE BOX OR FILL IN THE BLANK AS INDICATED.

For all questions, consider the above date when a Mayo Clinic staff member used a mobile device (eg, smartphone or tablet) to take photographs of you (or your child, if applicable). Unless otherwise specified, select one answer per question.

17-24 **1. Today's Date:** __ __/__ __/__ __ __ __
Month Day Year

25 **2. Do you recall a Mayo Clinic health care provider using a mobile device (phone, tablet, etc.) to take a photograph during a visit?**

1 ☐ No → **If no, do not answer further questions and return the survey.**
2 ☐ Yes

26 **3. What was the primary purpose of the photos?**

1 ☐ To send to a specialist to review → **Go to question 4 on page 2.**

2 ☐ To document what the photographed area looked like for future reference

3 ☐ In order to document the physical examination so that it did not have to be repeated by multiple providers

4 ☐ For educational purposes

5 ☐ Other, please specify: ______________

6 ☐ I am unsure why the photos were taken

(Options 2–6) → **Go to question 6 on page 2.**

27 **4. If you answered “To send to a specialist to review” for question 3 on page 2, why was it sent to a specialist?**

1 ☐ My provider knew what to diagnose me with but wanted a specialist’s assistance with management → **Go to question 6 below.**

2 ☐ My provider did not know what to diagnose me with and needed a specialist’s help in making a diagnosis → **Go to question 5 below.**

3 ☐ My provider thought sending the photo to a specialist would help me see a specialist sooner than if a photo had not been taken

4 ☐ Other, please specify: ______________________________

(Options 3 and 4) → **Go to question 6 below**

28 **5. If you answered “My provider did not know what to diagnose me with and needed a specialist’s help in making a diagnosis” for question 4 above, to what extent do you think photography affected the time until a final diagnosis was made and treatment was started (if treatment was necessary).**

1 ☐ Significantly delayed time to diagnosis and treatment

2 ☐ Slightly delayed time to diagnosis and treatment

3 ☐ Had no effect on time to diagnosis and treatment

4 ☐ Slightly expedited time to diagnosis and treatment

5 ☐ Significantly expedited time to diagnosis and treatment

29 **6. Were you asked for permission to take photographs?**

1 ☐ No, I was not specifically asked for permission

2 ☐ Yes, I was asked and gave verbal permission

3 ☐ Yes, I was asked to sign a form →

30 **If yes, did you read the entire form?**

1 ☐ No, I did not read any of the form

2 ☐ No, I only read part of the form

3 ☐ Yes, I read the entire from word-for-word

31 **7. How do you prefer to give permission for photographs to be taken and placed in your medical record when they are taken using a mobile device?**

1 ☐ I do not think specific permission is necessary
2 ☐ By telling my provider verbally
3 ☐ By signing a paper form
4 ☐ By signing the screen on the device used to take photos

32 **8. Rate your comfort level with photos being taken by your medical provider using a personal mobile device (such as an iPhone) compared to a clinic-owned camera.**

1 ☐ Strongly prefer personal mobile device
2 ☐ Slightly prefer personal mobile device
3 ☐ No preference
4 ☐ Slightly prefer clinic-owned camera
5 ☐ Strongly prefer clinic-owned camera

33 **9. Rate your comfort level with photos being taken by your medical provider compared to having a professional medical photographer take photos (regardless of the device used to take the photos).**

1 ☐ Strongly prefer professional photographer
2 ☐ Slightly prefer professional photographer
3 ☐ No preference
4 ☐ Slightly prefer provider
5 ☐ Strongly prefer provider

34 **10. Rate your comfort level with photos being available in your medical record, regardless of the method used to capture the photographs.**

1 ☐ Very uncomfortable
2 ☐ Somewhat uncomfortable
3 ☐ Not sure
4 ☐ Somewhat comfortable
5 ☐ Very comfortable

35 **11. Do you have concerns regarding the privacy or confidentiality related to the photos that were taken on the date noted at the top of page 1.**

1 ☐ No
36 2 ☐ Yes → **If yes, please explain:** ____

____

37 **12. Were security features of the application designed to keep photographs secure explained by the health care provider?**

1 ☐ No   2 ☐ Yes   3 ☐ Do not remember/unsure

13. Assuming that any information in the photos that could be used to personally identify you was removed, in which of the following settings would you feel comfortable having the photos that were taken shared? (Mark all that apply.)

38 1 ☐ One-on-one medical learner education
39 1 ☐ Large group (eg, classroom) medical learner education
40 1 ☐ Presentation at a national medical professional meeting
41 1 ☐ Publication in a medical journal or textbook
42 1 ☐ Publication on social media (eg, Twitter, blog) for medical education
43 1 ☐ None of the above — My photos should only be used for my medical care and not for education

44 **14. To what extent do you think photography affected the care you received?**

1 ☐ Very negatively affected my care
2 ☐ Somewhat negatively affected my care
3 ☐ Had no effect on my care
4 ☐ Somewhat positively affected my care
5 ☐ Very positively affected my care

45 **15. Do you own a phone with a camera?**

1 ☐ No   2 ☐ Yes

Go to question 16 on page 5.

46 **If yes, how interested would you be in the ability to transfer photos taken on your personal phone to your (or your child's) medical record?**

1 ☐ Not interested at all
2 ☐ Slightly interested
3 ☐ Not sure
4 ☐ Somewhat interested
5 ☐ Very interested

47 **Has a provider ever taken a photo of a phone screen to transfer a photo you (or someone accompanying you at the visit) had taken on a personal device into the medical record?**

1 ☐ No   2 ☐ Yes

48 **16. How likely would you be to recommend that a friend or family member give permission for use of medical photography using a mobile device in a similar clinical situation?**

1 ☐ Very unlikely
2 ☐ Fairly unlikely
3 ☐ Not sure
4 ☐ Fairly likely
5 ☐ Very likely

49 **17. In the future, if my provider recommended clinical photography during a visit, I would prefer:**

1 ☐ To not have photographs taken
2 ☐ For my health care provider to take photographs using his/her personal smartphone with a secure photo application
3 ☐ For my health care provider to take photographs using a clinic- or hospital-owned mobile device with a secure photo application
4 ☐ For my health care provider to take photographs using a clinic- or hospital-owned professional camera
5 ☐ For a professional medical photographer to take photographs using a clinic- or hospital-owned professional camera
6 ☐ I do not have a preference

50 **18. Please share any additional comments you have regarding your experiences with being photographed using a mobile device.**

Thank you for completing this survey!

©2017 Mayo Foundation for Medical Education and Research. All rights reserved. MAYO, MAYO CLINIC and the triple-shield Mayo logo are trademarks and service marks of MFMER.

MC4269-856
